# Supplementary figures and images for: Prognostic and Immunological Role of mRNA ac4C Regulator NAT10 in Pan-Cancer: New Territory for Cancer Research?
Source: Front Oncol. 2021 May 19;11:630417. doi: 10.3389/fonc.2021.630417 (PMC8170476; doi:10.3389/fonc.2021.630417)

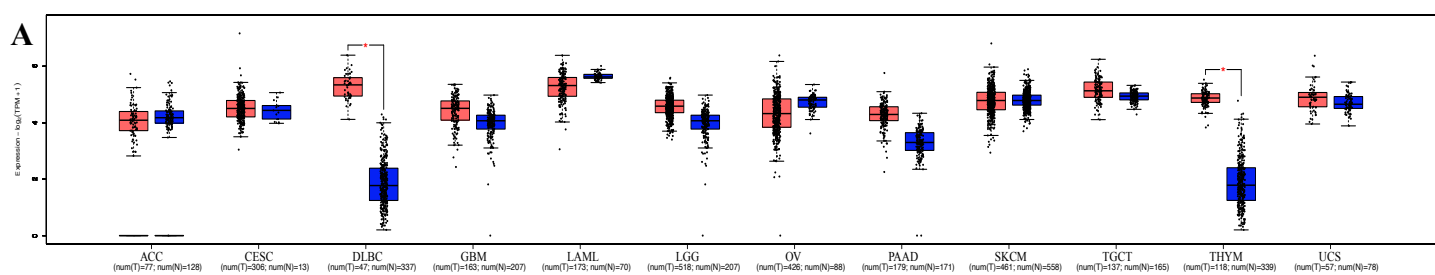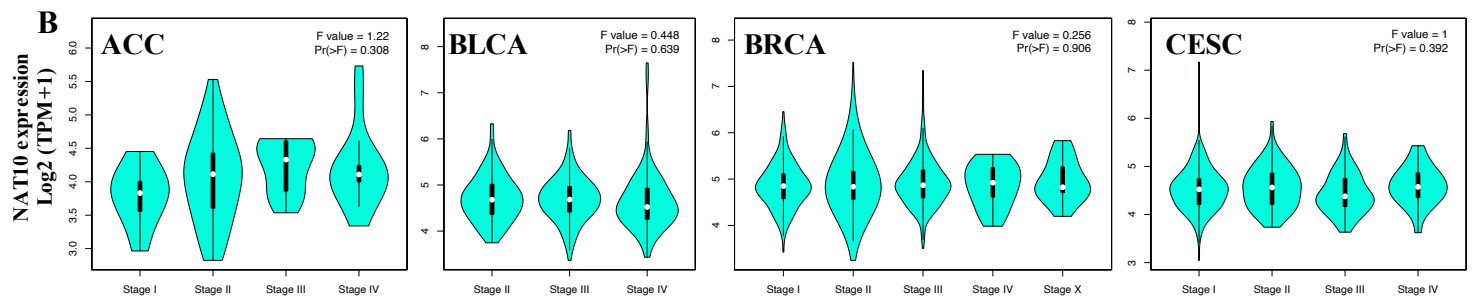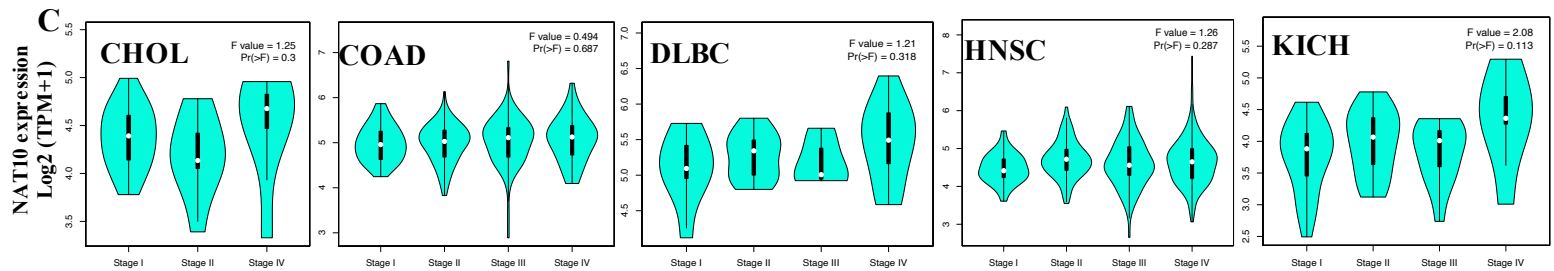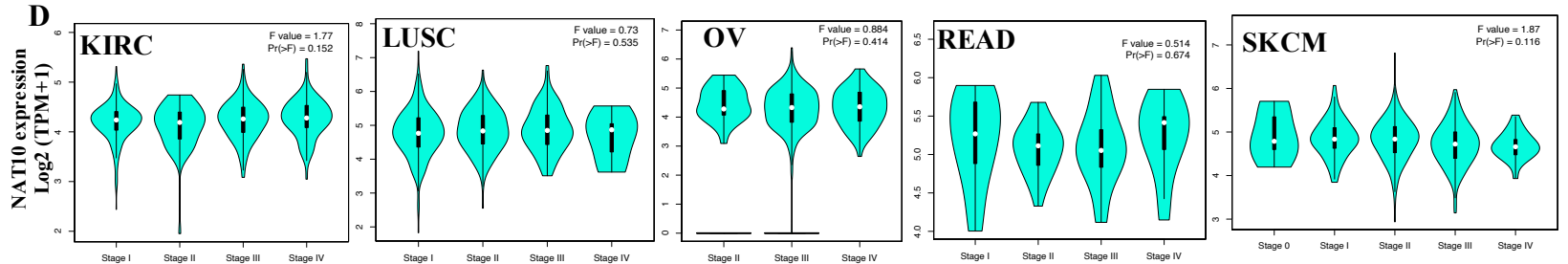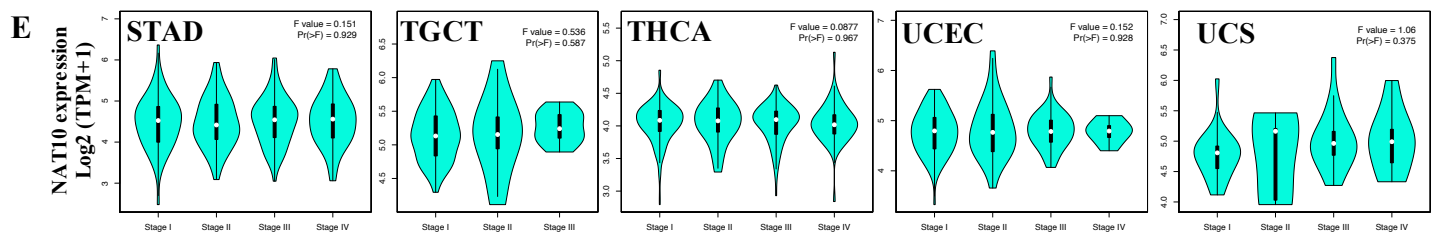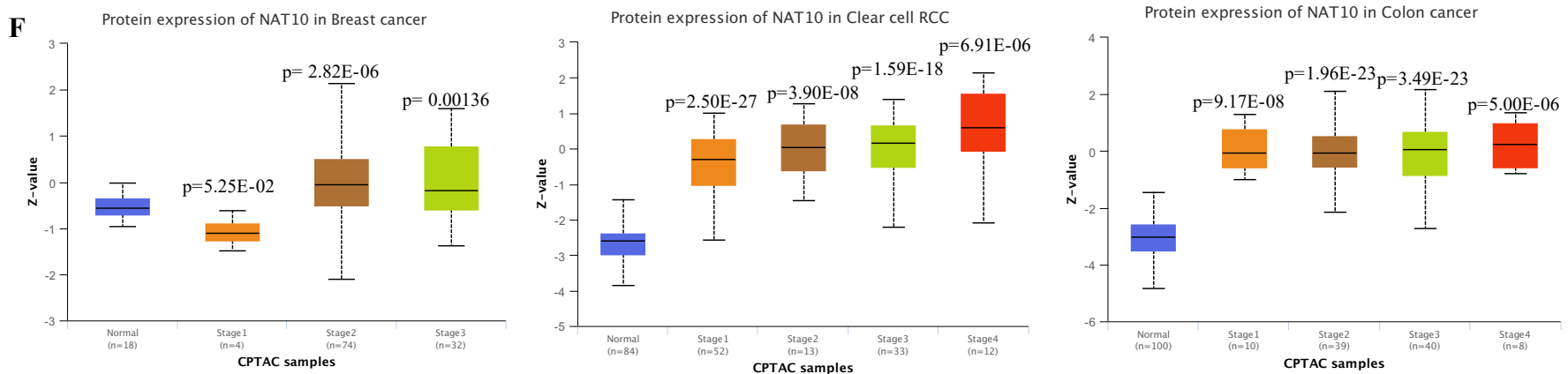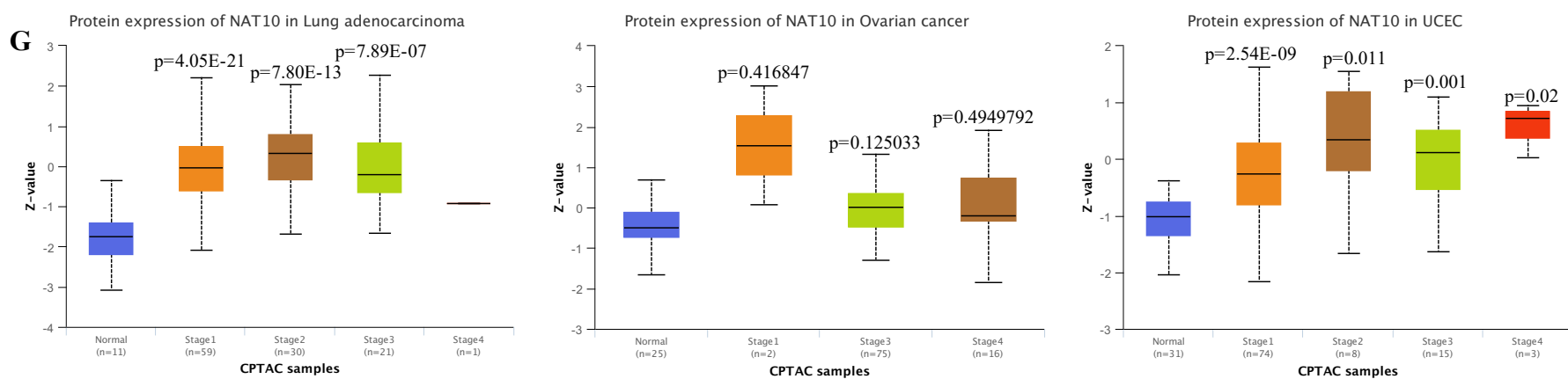

Supplement: Supplementary Figure 1 — Expression of the NAT10 gene in different tumors and pathological stages. (A) The expression statuses of the NAT10 gene in ACC, CESC, DLBC, GBM, LAML, LGG, OV, PAAD, SKCM, TGCT, THYM and UCS in TCGA project were compared with the corresponding normal tissues of the GTEx databases. (B–E) Expression of the NAT10 gene by different pathological stages of ACC, BLCA, BRCA, CESC (B), CHOL, COAD, DLBC, HNSC, KICH (C), KIRC, LUSC, OV, READ, SKCM (D), STAD, TGCT, THCA, UCEC and UCS (E). (E–G) Expression of the NAT10 total protein by different pathological stages of breast cancer, clear cell RCC, colon cancer (F), LUAD, ovarian cancer and UCEC (G). [file Image_1.pdf]

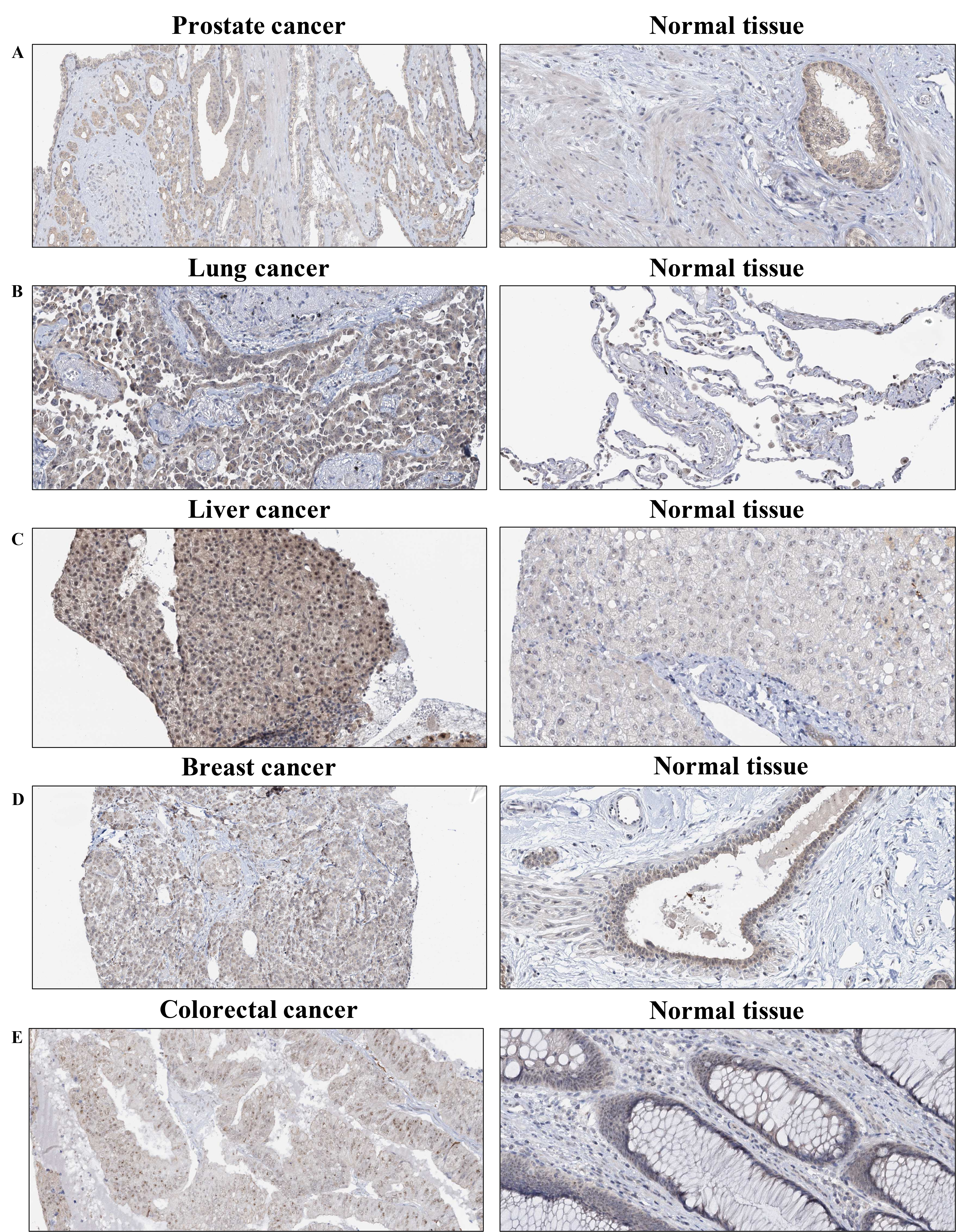

Supplement: Supplementary Figure 2 — The immunohistochemical staining from HPA database. (A–E) The immunohistochemical results from HPA database between prostate (A), lung (B), liver (C), breast (D) and colorectal cancer (E) with normal tissues. [file Image_2.jpeg]

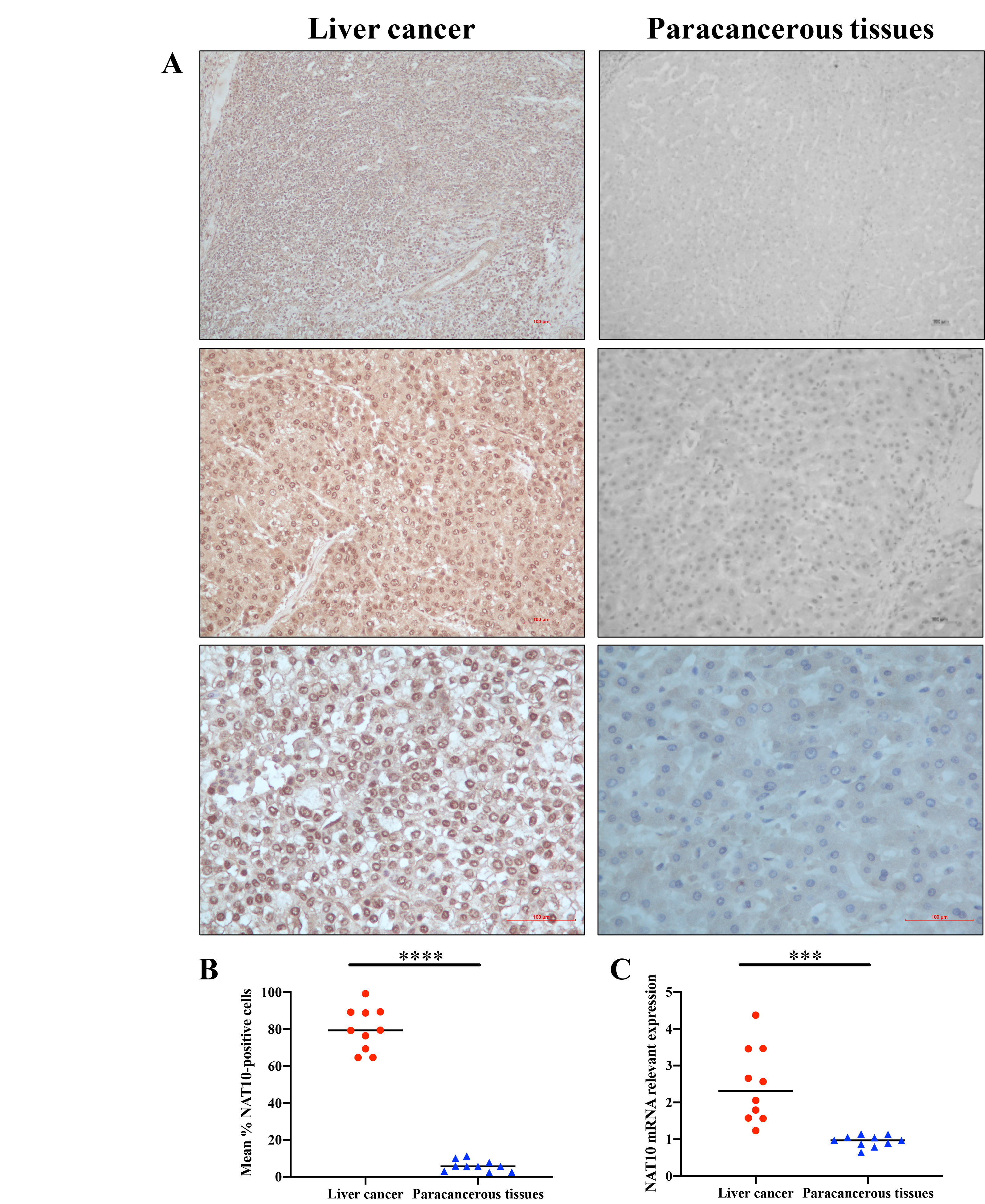

Supplement: Supplementary Figure 3 — NAT10 expression in LIHC and Paracancerous tissues from 10 patients. (A, B) The immunohistochemical (A) and mean % NAT10-positive cells results (B) in LIHC and Paracancerous tissues from 10 patients. (C) The NAT10 mRNA relevant expression in LIHC and Paracancerous tissues from 10 patients. [file Image_3.jpeg]

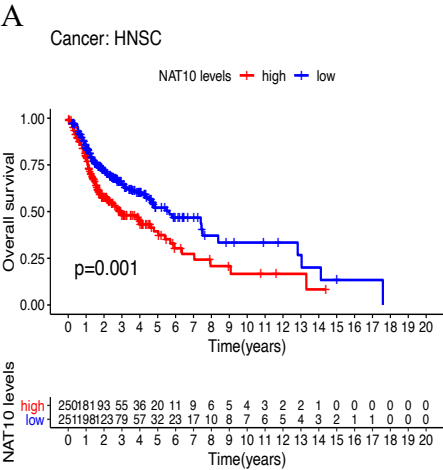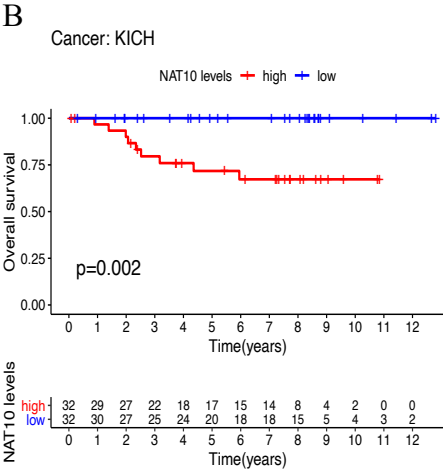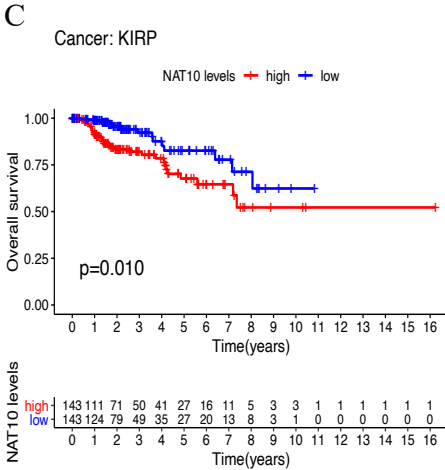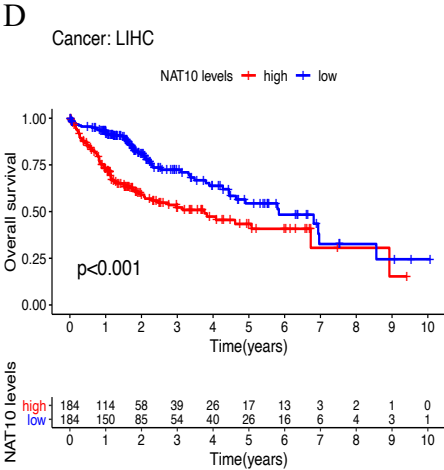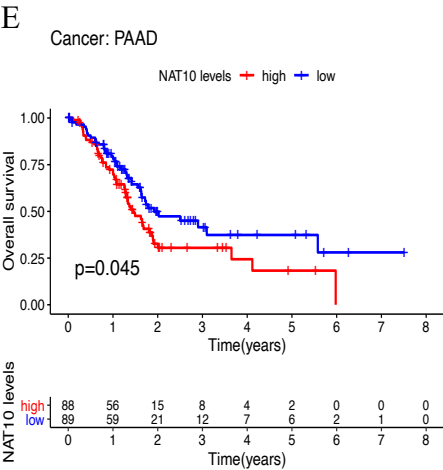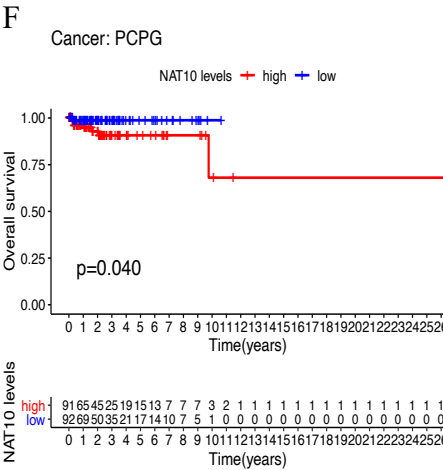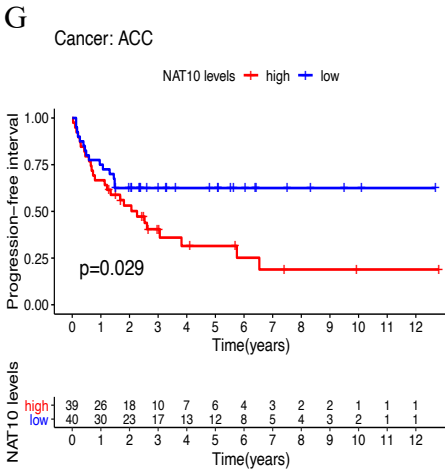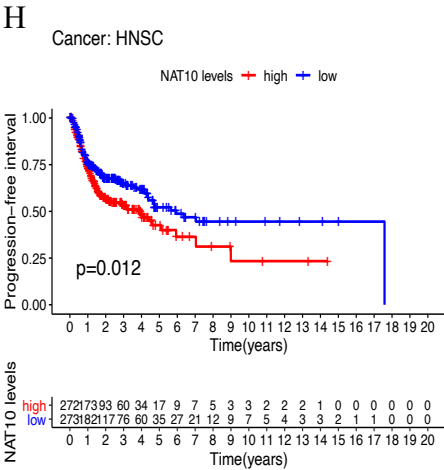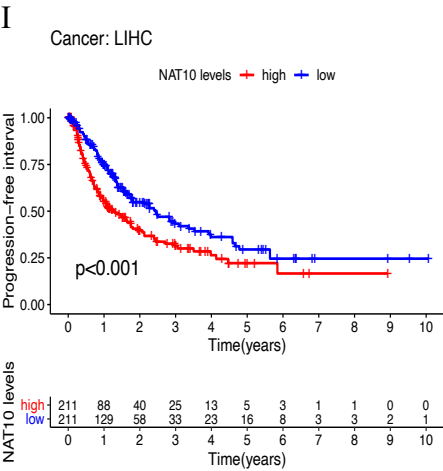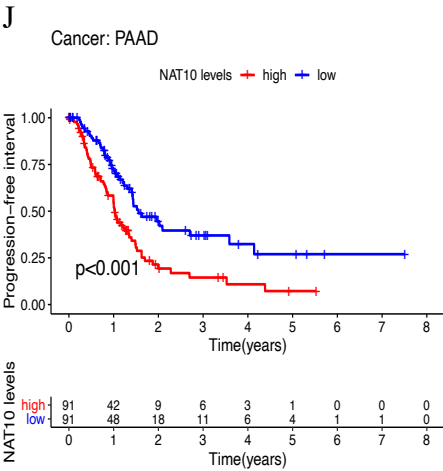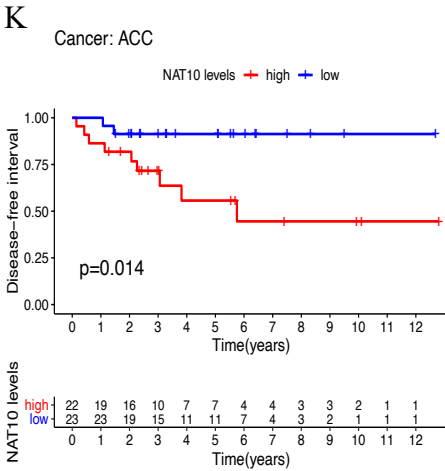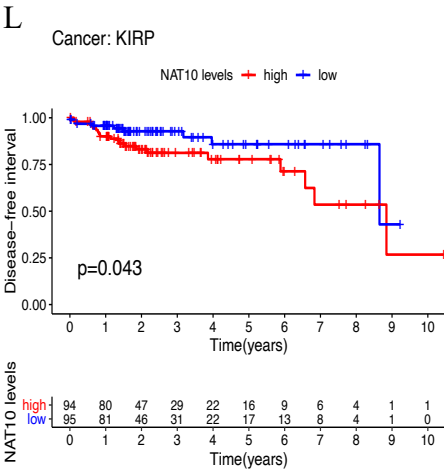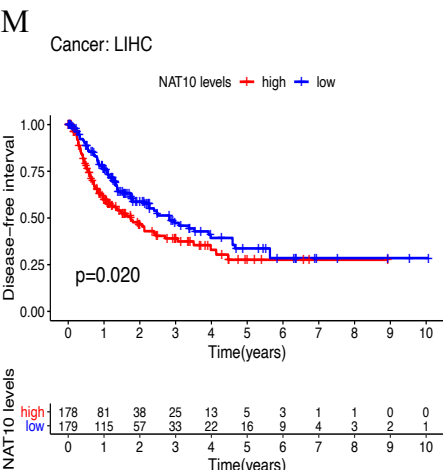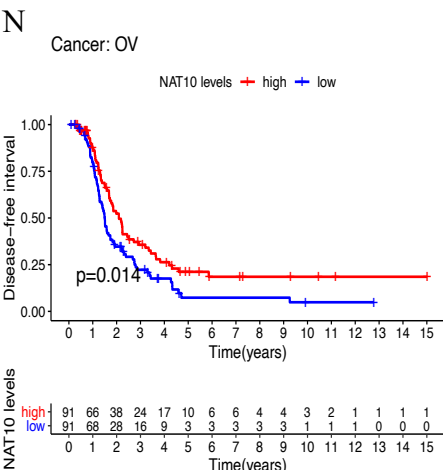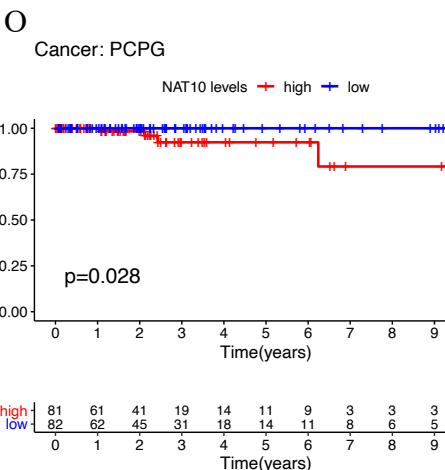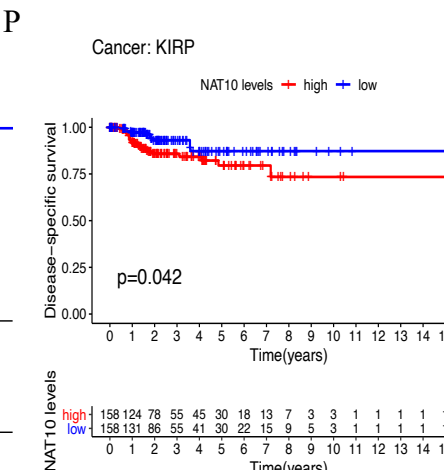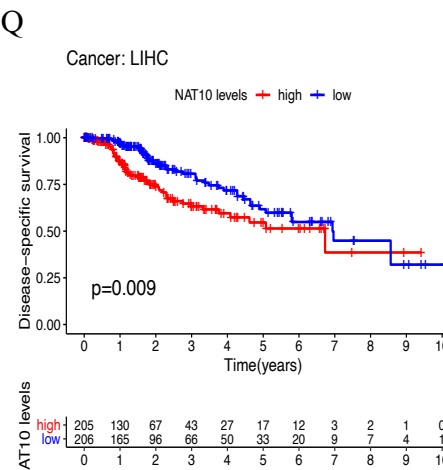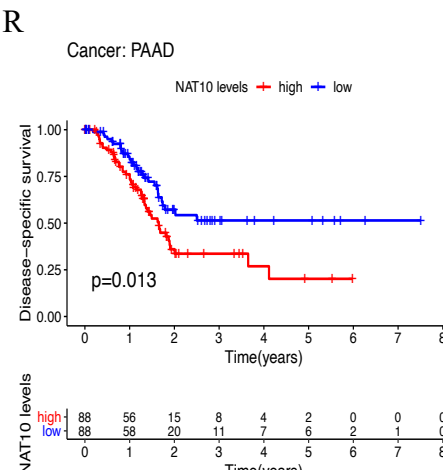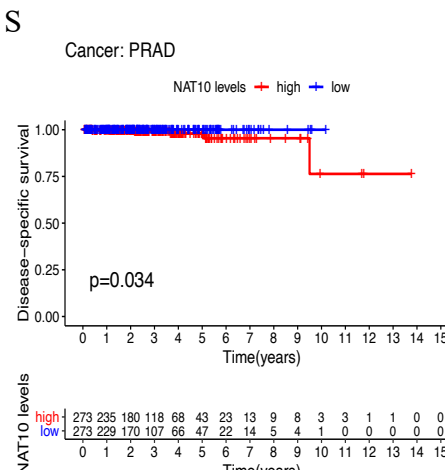

Supplement: Supplementary Figure 4 — Kaplan-Meier survival curves comparing high and low expression of NAT10 in different types of cancer in the TCGA database. (A–F) Survival curves for OS in six different cancers. (G–J) Survival curves for progression-free interval (PFI) in four different cancers. (K–O) Survival curves for disease-free interval (DFI) in five different cancers. (P–S) Survival curves for DSS in four different cancers. Red curve represents patients with high expression of NAT10. [file Image_4.pdf]

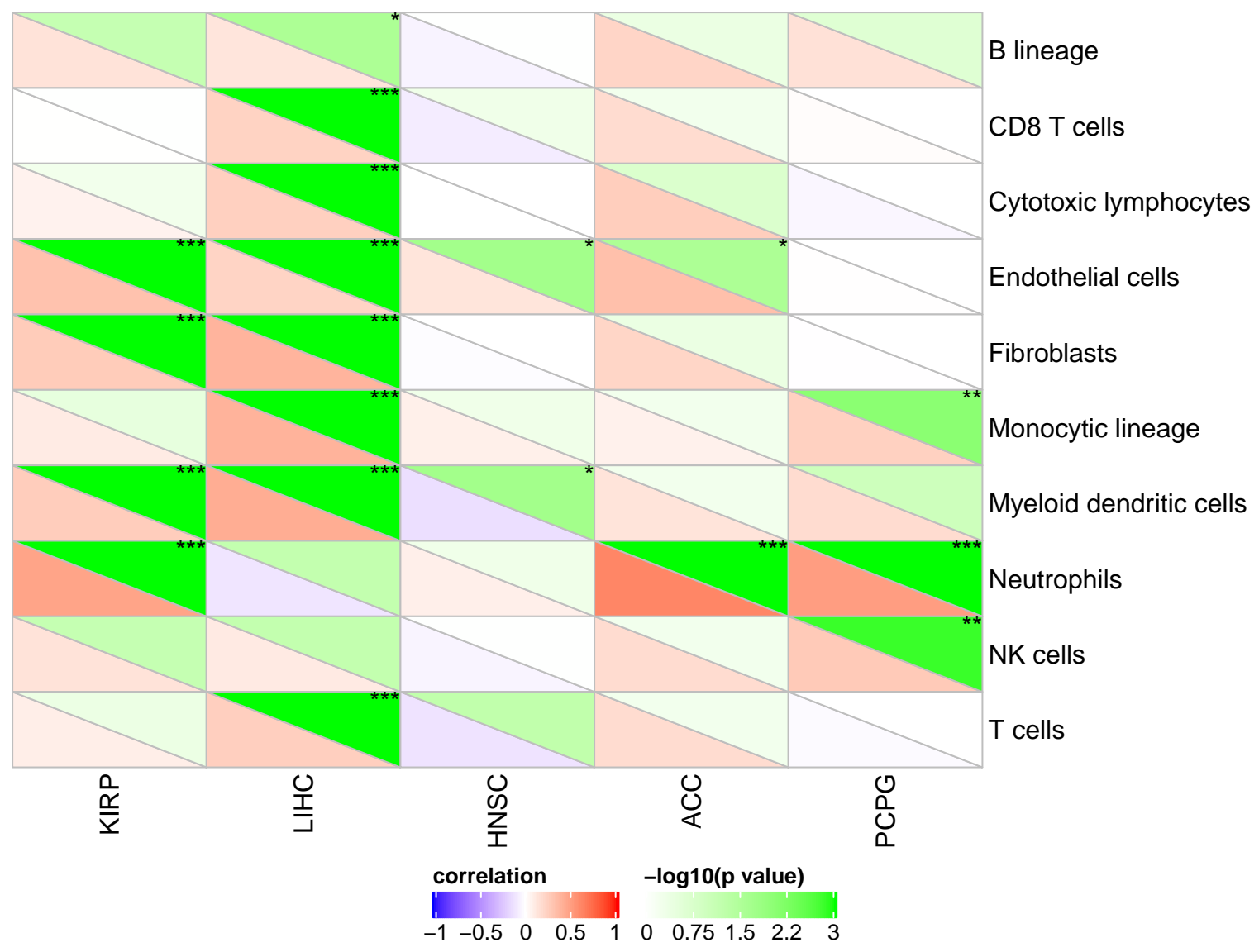

Supplement: Supplementary Figure 5 — Correlation between NAT10 expression with immune infiltration level in MCPcounter. The lower triangle in each tile indicates coefficients calculated by Pearson’s correlation test, and the upper triangle indicates log10-transformed P-values. [file Image_5.pdf]
